# Insole-pressure distribution in three pressure-relief postoperative shoes

**DOI:** 10.1186/1757-1146-7-S1-A10

**Published:** 2014-04-08

**Authors:** Paolo Caravaggi, Alessia Giangrande, Lisa Berti, Sandro Giannini, Carlo Ferraresi, Alberto Leardini

**Affiliations:** 1 Movement Analysis Laboratory, Istituto Ortopedico Rizzoli, Bologna, 40136, Italy; 2 1st Division of orthopedic surgery, Istituto Ortopedico Rizzoli, Bologna, 40136, Italy; 3 DIMEAS, Politecnico di Torino, Torino, 10129, Italy

## Background

Patients undergoing forefoot surgery often require specific footwear to relieve the operated area. Post-operative footwear can be very different in relation to the amount of weight born by the forefoot [1,2]. While these special shoes are intended to be worn for short periods following surgery, a compromise must be found between level of comfort and functionality. In this study the insole-pressure distribution of two shoes, specifically designed to unload the forefoot, was compared to that of a comfortable shoe manufactured by the same company.

## Material and methods

10 healthy female subjects (28.2 ± 10.0 yrs, 1.64 ± 0.04 m, 55.1 ± 3.7 kg) were asked to walk at comfortable speed wearing three shoe types produced by the same company: WPS^®^, TD^®^ and Deambulo^®^ (Podartis, Treviso, Italy). WPS^®^ and TD^®^ are post-op shoes designed explicitly for forefoot off-loading, whereas Deambulo^®^ is meant to be worn in the rehabilitation period, about 20/30 days after surgery. An insole pressure measurement system (Pedar X, Novel GmbH, Munich, Germany) was employed to record plantar pressure. Several walking trials were recorded for each subject in three configurations of shoes for left and right foot respectively: WPS^®^ + Deambulo^®^, TD^®^ + Deambulo^®^ and Deambulo^®^ + Deambulo^®^. The configurations were established to be consistent with the post-op clinical course when patients wear the pressure-relief shoe on the operated side, and a more comfortable one on the contralateral foot. Mean and peak pressure, vertical force, force and pressure time integral, were recorded for different regions of the insole together with spatiotemporal parameters. For each subject in each configuration, three consistent steps from the same trial were used in the analysis.

## Results

**Figure 1:**
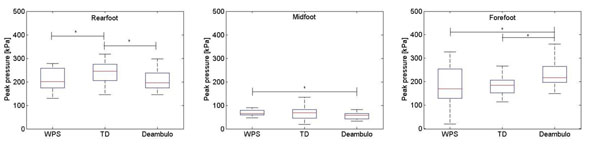
From left to right: boxplot of the peak pressure at the rearfoot, midfoot and forefoot across 30 samples for each shoe group. * denotes statistically significant difference (p<0.05) between groups.

Good consistency in the baropodometric measurements was found in each group across all subjects. No difference was detected in walking speed between the three shoe groups. Both WPS^®^ and TD^®^ helped decrease the peak pressure at the forefoot (Fig. 1), with much less variability over subjects in the TD^®^ group. In general, the WPS^®^ showed the smallest pressure and force values under the forefoot and the largest at the midfoot, especially when compared to the Deambulo^®^. At the rearfoot, force and pressure were the largest in the TD^®^ group.

## Conclusions

Both WPS^®^ and TD^®^ helped decrease forefoot pressure thus are indicated for the postoperative course of patients undergoing forefoot surgery. Some load compensation with larger pressures at either the midfoot or rearfoot has been revealed respectively in these two shoes.
